# Effects of prolonged exercise versus multiple short exercise sessions on risk for metabolic syndrome and the atherogenic index in middle-aged obese women: a randomised controlled trial

**DOI:** 10.1186/s12905-017-0421-z

**Published:** 2017-08-22

**Authors:** JinWook Chung, KwangJun Kim, Jeeyoung Hong, Hyoun-Joong Kong

**Affiliations:** 1 Sport Culture Science Department, Dongguk University-Seoul, 30, Pildong-ro 1-gil, Jung-gu, Seoul, 04620 Republic of Korea; 2 Sports Science Department, Korea Instiute of Sports Science, 727 Hwarang-ro, Nowon-gu, Seoul, 01794 Republic of Korea; 3 Biomedical Research Institute, Seoul National University Hospital, 101 Daehak-Ro, Jongno-gu, Seoul, 03080 Republic of Korea; 4 Institute of Medical & Biological Engineering, Medical Research Center, College of Medicine, Seoul National University, 71 IhwaJang-gil, Jongno-gu, Seoul, 03087 Republic of Korea; 5 Department of Biomedical Engineering, College of Medicine, Chungnam National University, Munhwa-ro 266, Jung-gu, Daejeon, 35015 Republic of Korea; 6 Department of Biomedical Engineering, Chungnam National University Hospital, Munhwa-ro 282, Jung-gu, Daejeon, 35015 Republic of Korea

**Keywords:** Intermittent exercise, Prolonged exercise, Metabolic syndrome, Arteriosclerosis, Atherogenic index, Obesity, Women

## Abstract

**Background:**

Many people, although they may recognise the positive effects of exercise, do not exercise regularly owing to lack of time. This study aimed to investigate the effects of prolonged single-session exercise and multiple short sessions of exercise on the risk of metabolic syndrome and the atherogenic index in middle-aged obese women.

**Methods:**

Thirty-six participants were divided into the single-session group, multiple-session group, and control group. The single-session group engaged in one session of treadmill exercise for 30 min a day; the multiple-session group had three sessions of 10 min a day. Both groups exercised 3 days/week for 12 weeks. The control group did not perform any exercise.

**Results:**

The single-session group showed decreases in weight (0.97 kg [95% C.I. = 0.09–1.83], *p* < .05), body mass index (0.43 kg/m^2^ [95% C.I. = 0.03–0.81], *p* < .05), and fat mass (1.65 kg, [95% C.I. = 0.78–2.51], *p* < .01). Systolic blood pressure dropped in the single-session group (6.66 mmHg, [95% C.I. = 1.44–11.88], *p* < .05), and diastolic blood pressure dropped in the multiple-session group (3.38 mmHg, [95% C.I. = 1.44–5.88], *p* < .01). High-density lipoprotein cholesterol rose in the single-session group (4.08 mg/dL, [95% C.I. = −8.08–(−)0.07], *p* < .05) and dropped in the control group (10.75 mg/dL [95% C.I. = 1.95–19.54], *p* < .01). According to post hoc analysis, high-density lipoprotein cholesterol increased more in the single-session group than the control group (95% C.I. = 0.61–21.88, *p* < .05). Glucose levels decreased in both the single-session group (16 mg/dL [95% C.I. = 5.64–26.35], *p* < .01) and the multiple-session group (12.16 mg/dL, [95% C.I. = 2.18–22.14], *p* < .05). Waist circumference decreased in the single-session group (2.65 cm [95% C.I. = 1.46–3.83], *p* < .001) and multiple-session group (2.04 cm, [95% C.I. = 1.51–2.73], *p* < .001). Low-density lipoprotein cholesterol levels rose in both the multiple-session group (−15.79 mg/dL [95% C.I. = −34.24–(−)3.78], *p* < .05) and the control group (−22.94 mg/dL [95% C.I. = −44.63–(−)1.24], *p* < .05). The atherogenic index increased in the control group (−1.06 [95% C.I. = −1.69–(−)0.41], *p* < .01).

**Conclusions:**

The findings indicate that prolonged exercise is superior to multiple short sessions for improving the risk of metabolic syndrome and the atherogenic index in middle-aged obese women. However, multiple short sessions can be recommended as an alternative to prolonged exercise when the goal is to decrease blood glucose or waist circumference.

## Background

Metabolic syndrome and atherosclerosis are problematic because of their association with ensuing cardiovascular disease (CVD), and their prevalence has been increasing [1, 2]. A high cholesterol level increases the risk of developing cardiovascular disease. The atherogenic index (AI) has recently started to gain attention as an important indicator of the risk of CVD and atherosclerosis [3] and is more useful in CVD risk prediction than lipid concentrations [4]. The risk of atherosclerosis increases when the AI is over 5.0 for men and 4.0 for women [5]. These problems arise more frequently in obese individuals and generally result from an unrestricted-calorie diet and lack of physical activity [6–8].

Middle-aged persons are usually confronted with various physiological, physical, cognitive, and social changes that make them vulnerable to chronic disease. Women have particularly high morbidity rates in midlife, as menopause changes their hormonal profile, blood pressure, blood lipid levels, and body fat distribution in ways that may increase the risk of metabolic syndrome and atherosclerosis [9, 10]. Indeed, in middle-aged women, the risk of cardiovascular disease increases by 50% after menopause, making it of the utmost importance to live a healthy lifestyle and eliminate risk factors such as physical inactivity [11–13].

Treatment regimens for obesity include a calorie-restricted diet [14] and exercise [15] and may involve drugs or surgery. Exercise is especially known as an economic and effective approach to reduce fat accumulation and promote physical strength [16]. Regular and continuous physical activity helps control obesity, reduces the risk of metabolic syndrome, and lowers the AI, thus helping prevent or postpone the development of cardiovascular disease in obese individuals [17, 18].

However, many people, despite recognising the positive effects of exercise, are unable to exercise regularly or for prolonged periods, owing to their busy schedules. Therefore, the American College of Sports Medicine and Centers for Disease Control and Prevention, while recommending that all adults engage in 30 min of moderate exercise every day, suggests that this can be done in multiple short sessions, as opposed to the traditional approach of one long session [19]. Hardman [20] reported that several short exercise sessions resulted in as much improvement in physical strength as a single long session. Miyashita et al. [21] found that ten 3-min bouts of exercise lowered blood lipid levels as much as one 30-min bout in healthy men. Gayda et al. [22] reported that multiple short exercise sessions had similar effects on fat metabolism as a single long session in 18 patients with chronic heart failure.

Although several prior studies have emphasised the importance of short exercise sessions, no study has statistically evaluated short-term changes in risk for metabolic syndrome and AI in middle-aged obese women by comparing single prolonged exercise sessions and multiple short exercise sessions. Thus, it was the objective of this study to compare the effects of a single long exercise session and multiple short exercise sessions on the risk of metabolic syndrome and the AI in middle-aged obese women. Specifically, we focused on analysing the effectiveness of multiple short sessions of exercise.

## Methods

### Participants

Participants for this randomised controlled trial were recruited by a research coordinator from among 55 middle-aged women with a body fat percentage of 30% or higher [23] who participated in a voluntary exercise class at the Yeonsu-Gu public health centre in Incheon, South Korea. We assessed the eligibility of all participants. Eight individuals were excluded on the basis of the following criteria: engaging in regular exercise over the previous 6 months or an inability to perform exercise due to motor malfunctions, unstable cardiovascular diseases, diabetes mellitus, mental illness, or personal reasons.

**Fig. 1:**
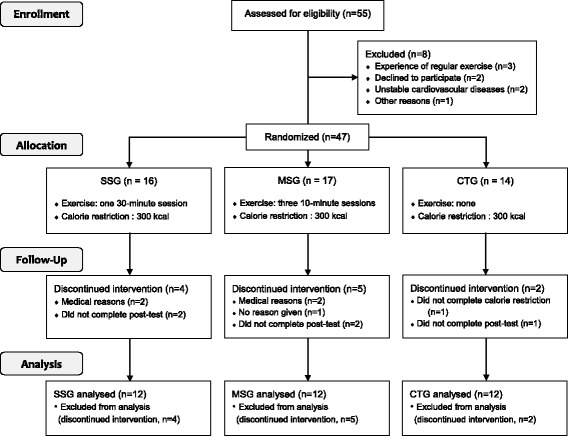
Flowchart of the study design

Participants were assigned to engage in a single long session of exercise (single-session group, SSG; *n* = 16), multiple short sessions (multiple-session group; MSG, *n* = 17), or no exercise (control group, CTG; *n* = 14) with block random sampling by the research coordinator. Both groups were blinded after assignment of the interventions. Over the course of the study, 11 participants dropped out for medical or personal reasons. Thirty-six women completed the study and were included in the final analysis sample. The exercise intervention and outcome measurement were conducted at the same public health centre. Figure 1 illustrates the study design and detailed course of the study. The physical characteristics of the participants are presented in Table 1.

**Table 1:** Physical characteristics of participants

	SSG (*n* = 12)	MSG (*n* = 12)	CTG (*n* = 12)	F	*p*
Age (years)	49.33 ± 5.06	47.75 ± 4.59	49.55 ± 4.29	.515	.602
Height (cm)	154.75 ± 6.38	155.55 ± 5.70	154.50 ± 5.76	.102	.904
Weight (kg)	60.42 ± 9.22	59.45 ± 5.38	60.62 ± 6.79	.087	.917
Fat percentage (%)	36.95 ± 4.22	34.65 ± 3.94	35.95 ± 5.32	.775	.469
BMI (kg/m^2^)	25.27 ± 3.94	24.57 ± 1.76	25.39 ± 2.50	.282	.756
Fat mass (kg)	22.53 ± 5.38	20.63 ± 3.12	21.83 ± 4.8	.538	.589

*SSG* single-session group, *MSG* multiple-session group, *CTG* control group, *BMI* body mass index

Values are presented as mean ± SD; *P* values represent one-way ANOVA

To check whether the sample would produce statistically valid results, G*power software [24] was used to estimate the proper sample size given an effect size of 0.30 (for percent body fat), an α value (probability of a Type 1 error) of 0.05, and a desired statistical power (1-β) of 0.85. Thirty-six participants were needed according to the software’s calculations.

The study was carried out in accordance with the Declaration of Helsinki and was approved by the Institutional Review Board of Korea Institute of Sport Science. Written informed consent was obtained from all participants, who were all aware of the nature of the study.

### Exercise programmes

The exercise programme was performed 3 days a week according to the recommendation of the U.S. Department of Health and Human Services [25]. Both groups exercised on a treadmill 3 days a week for 12 weeks supervised by an exercise specialist. On workout days, the SSG engaged in one 30-min session of exercise, whereas the MSG engaged in three 10-min sessions with about 4 h between sessions. The programme was designed to expend 200 kcal in 30 min, with the treadmill speed and grade determined based on the relationship between oxygen consumption (VO_2_) and calorie expenditure (Eqs. 1 and 2) [17].

1 $$ {\mathrm{VO}}_2\left(\mathrm{L}/\min \right)=\mathrm{calorie}\  \mathrm{expenditure}\ \left(\mathrm{kcal}/\min \right)/\left(5\ \mathrm{L}/\mathrm{kcal}\right) $$

2 $$ {\mathrm{VO}}_2\left(\mathrm{mL}/\mathrm{kg}/\min \right)=\left(3.5\ \mathrm{mL}/\mathrm{kg}/\min \right)+\left(0.2\times \mathrm{speed}\right)+\left(0.98\times \mathrm{speed}\times \mathrm{grade}\right) $$

To determine the appropriate intensity of exercise necessary for each participant to expend 200 kcal in 30 min, the SSG and MSG underwent a submaximal exercise test using a metabolic gas analysis system (Quark b^2^, COSMED, Rome, Italy) to measure their maximum oxygen consumption (VO_2max_). The Bruce protocol was applied for the test. The test ended when the participant reached 85% of maximum heart rate or exhaustion, whichever came first. The maximal heart rate was calculated by subtracting each participant’s age from 220 (age-predicted maximal heart rate) [26]. The oxygen consumption of each participant was set to 83.12% (±3.93%) of VO_2max_.

### Calorie restriction

All participants were instructed to reduce their usual intake by 300 kcal and to keep a food diary to assist them in complying with the restriction. Based on the food diary, the mean daily calorie intake was calculated by using nutrition analysis software (CAN-pro 2.0, The Korean Nutrition Society, Seoul, Korea) once a week. A professional nutritionist provided nutrition education and counselling every 4 weeks. They were also required to keep a daily expenditure diary to maintain their usual activity or exercise routine. The exercise specialist monitored their daily expenditure each week to verify that they maintained their usual activity routine. Both food and daily expenditure diaries were only used for monitoring purposes. The energy deficit from exercise in this study was set to 200 kcal, which resulted in a 500-kcal deficit on exercise days in the exercise groups [27].

### Outcome measures

To examine changes of the body composition, metabolic syndrome risk factors and arteriosclerosis-related factors, a pre-test was done one week before the application of the exercise program and a post-test was conducted during the last week of the 12-week program.

### Body composition

Bioelectrical impedance analysis (BIA) was conducted to measure weight, body mass index (BMI), fat-free mass, fat mass, and fat percentage (InBody 720, Inbody Co., Seoul, Korea). The participants were instructed to fast for 4 h, rest for 13 h without exercise, and urinate 30 min prior to the measurement. Whole-body BIA measurements were taken with the participant standing upright with bare feet on the scale and arms abducted 90° while holding the handle of the device [28, 29].

### Risk factors for metabolic syndrome

To assess participants’ risk of developing metabolic syndrome, the following measurements were taken: blood pressure (BP), serum high-density lipoprotein (HDL) level, serum triglyceride (TG) level, blood glucose level, and waist circumference. Systolic and diastolic BP (SBP and DBP, respectively) were measured with an automatic BP monitor (Easy X 800, Jawon Medical Co., Kyungsan, Korea) after a minimum of 5 min of rest. To measure HDL, TG, and glucose levels, 5 mL or more blood was extracted from the brachial veins with a disposable syringe; samples were taken after at least 9 h of fasting, centrifuged for about 10 min at 3000 rpm to separate serum, cryogenically processed, and sent to a laboratory specialising in blood analysis. Pre-test samples (the day before training) and post-test samples (the day after training) were obtained in the morning. Waist circumference was measured at the halfway point between the lower rib and the ilium ridge, as recommended by the World Health Organization [30].

### Arteriosclerosis-related factors

Blood levels of cholesterol (total cholesterol, TC) and low-density lipoprotein (LDL) were measured. The AI – an indicator of the risk of coronary artery disease [31] – was calculated by using the following formula: (TC - HDL)/HDL [32].

### Statistical analyses

All statistical analyses were performed using SPSS 20.0 (IBM Co., Armonk, NY, USA). The Kolmogorov-Smirnov one-sample test was used to determine whether the study variables were normally distributed, and normal distribution was confirmed for all variables. Therefore, a two-way repeated measure ANOVA was performed to assess changes in and interactions among the variables over time for the three groups. When there were significant interaction effects and main effects of time, contrast analysis was used to examine pre- and post-test differences in each group. When there were significant main group effects, Tukey’s post hoc analysis was used to examine the differences in each group. A one-way ANOVA was used to test the homogeneity of physical characteristics among the three groups before the intervention. The level of statistical significance was set at α = .05 for all tests.

## Results

### Physical characteristics of participants

There were no significant differences among the groups in height, weight, fat percentage, BMI, or fat mass before the intervention (Table 1).

### Body composition

Table 2 shows changes in body composition after the exercise programmes. Two-way repeated-measure ANOVA revealed time of measurement to have significant main effects on weight (*p* < .05), BMI (*p* < .05) and fat mass (*p* < .05). For fat mass, there was also a significant interaction effect between group and time (*p* < .001). Contrast analysis revealed significant reductions in weight (0.97 kg [95% C.I. = 0.09–1.83], *p* < .05), body mass index (0.43 kg/m^2^ [95% C.I. = 0.03–0.81], *p* < .05), and fat mass (1.65 kg, [95% C.I. = 0.78–2.51], *p* < .01) in the single-session group after intervention.

**Table 2:** Changes in body composition

Variable	SSG (*n* = 12)		MSG (*n* = 12)		CTG (*n* = 12)	
	Before	After	Before	After	Before	After
Weight (kg)^†^	60.43 ± 9.23	59.46 ± 9.32^‡^	59.46 ± 5.39	58.75 ± 5.30	60.63 ± 6.79	60.81 ± 6.62
BMI (kg/m^2^)^†^	25.28 ± 3.94	24.85 ± 3.82^‡^	24.58 ± 1.76	24.30 ± 1.95	25.39 ± 2.51	25.46 ± 2.35
Lean mass (kg)	20.38 ± 2.82	20.73 ± 3.26	20.89 ± 2.36	20.67 ± 2.14	20.78 ± 2.24	20.87 ± 2.51
Fat mass (kg)^*, †^	22.53 ± 5.38	20.88 ± 4.96^‡‡^	20.63 ± 3.13	20.36 ± 3.54	21.83 ± 4.80	22.39 ± 4.39
Fat percentage (%)	36.95 ± 4.23	34.88 ± 4.06	34.65 ± 3.95	34.55 ± 4.44	35.95 ± 5.32	36.73 ± 5.48

Values are presented as mean ± SD

*SSG* single-session group, *MSG* multiple-session group, *CTG* control group, *BMI* body mass index

^*^ Significant interaction effect between time and group (*p* < .001)

^†^ Significant main effect of time (*p* < .05)

^‡, ‡‡^ Significantly different from pre-intervention value (*p* < .05 and *p* < .01, respectively)

### Risk factors for metabolic syndrome

Table 3 shows changes in factors affecting the risk of metabolic syndrome before and after the intervention. Measurement time had significant main effects on systolic blood pressure (*p* < .01), diastolic blood pressure (*p* < .05), and glucose (*p* < .001). For waist circumference, both the main effect of measurement time (*p* < .001) and the interaction effect between time and group (*p* < .001) were significant. For high-density lipoprotein cholesterol, there was a significant main effect of group (*p* < .05), as well as an interaction effect between time and group (*p* < .01). According to the Tukey’s post hoc analysis, high-density lipoprotein cholesterol increased more in the single-session group than in the control group (*p* < .05). High-density lipoprotein cholesterol increased in the single-session group (4.08 mg/dL, [95% C.I. = −8.08–(−)0.07], *p* < .05) and decreased in the control group (10.75 mg/dL [95% C.I. = 1.95–19.54], *p* < .01). Contrast analysis results showed a decrease in systolic blood pressure (6.66 mmHg, [95% C.I. = 1.44–11.88], *p* < .05) in the single-session group and a reduction in diastolic blood pressure (3.38 mmHg, [95% C.I. = 1.44–5.88], *p* < .01) in the multiple-session group. Serum glucose levels and waist circumference decreased in both the single-session group (16 mg/dL [95% C.I. = 5.64–26.35], *p* < .01 and 2.65 cm [95% C.I. = 1.46–3.83], *p* < .001 respectively) and multiple-session group (12.16 mg/dL, [95% C.I. = 2.18–22.14], *p* < .05 and 2.04 cm [95% C.I. = 1.51–2.73], *p* < .001, respectively).

**Table 3:** Changes in factors associated with metabolic syndrome

Variable	SSG (*n* = 12)		MSG (*n* = 12)		CTG (*n* = 12)	
	Before	After	Before	After	Before	After
SBP (mm Hg)^††^	142.33 ± 13.90	135.67 ± 10.76^‡^	131.54 ± 11.53	129.31 ± 7.86	136.58 ± 14.20	136.33 ± 12.65
DBP (mm Hg)^†^	88.00 ± 11.96	84.33 ± 6.67	81.00 ± 6.29	77.62 ± 5.30^‡‡^	84.92 ± 10.47	85.08 ± 9.05
HDL (mg/dL)^*, §, 1>3^	61.50 ± 16.38	65.58 ± 13.51^‡^	54.38 ± 9.95	55.85 ± 8.22	57.67 ± 13.20	46.92 ± 9.89^‡‡^
TG (mg/dL)	115.58 ± 52.99	110.50 ± 51.15	99.75 ± 28.06	93.00 ± 27.97	100.58 ± 32.44	100.46 ± 23.64
Glucose (mg/dL)^†††^	111.08 ± 32.44	95.08 ± 22.82^‡‡^	98.58 ± 15.34	86.42 ± 6.41^‡^	92.42 ± 11.61	87.42 ± 6.52
Waist circumference (cm)^**,†††^	81.08 ± 9.44	78.43 ± 9.46^‡‡‡^	77.62 ± 5.20	75.58 ± 5.45^‡‡‡^	80.25 ± 5.91	80.75 ± 5.87

Values are presented as mean ± SD

*SSG* single-session group, *MSG* multiple-session group, *CTG* control group, *BMI* body mass index, *SBP* systolic blood pressure, *DBP* diastolic blood pressure, *HDL* high-density lipoprotein

^*, **^ Significant interaction effect between time and group (*p* < .01 and *p* < .001, respectively)

^†, ††, †††^ Significant main effect of time (*p* < .05, *p* < .01, and *p* < .001, respectively)

^§^ Significant main effect of group (*p* < .05, 1: SSG, 3: CTG)

^‡, ‡‡, ‡‡‡^ Significantly different from pre-intervention value (*p* < .05, *p* < .01, and *p* < .001, respectively)

### Arteriosclerosis-related factors

Table 4 shows changes in the arteriosclerosis-related factors before and after the intervention. For low-density lipoprotein cholesterol levels, there was an interaction effect (*p* < .05) between time and group. For AI, both the main effect of measurement time (*p* < .05) and the interaction effect between time and group (*p* < .01) were significant. Contrast analysis showed increases in low-density lipoprotein cholesterol levels in both the multiple-session group (−15.79 mg/dL, [95% C.I. = −34.24–(−)3.78], *p* < .05) and the control group (−22.94 mg/dL [95% C.I. = − 44.63–(−)1.24], *p* < .05). The AI increased in the control group (−1.06 [95% C.I. = −1.69–(−)0.41], *p* < .01).

**Table 4:** Changes in factors related to atherogenic index

Variable	SSG (*n* = 12)		MSG (*n* = 12)		CTG (*n* = 12)	
	Before	After	Before	After	Before	After
TC (mg/dL)	188.75 ± 26.02	183.75 ± 23.11	184.15 ± 31.14	202.31 ± 21.52	173.58 ± 37.91	185.75 ± 29.39
LDL (mg/dL) ^*, †^	106.70 ± 26.61	96.07 ± 27.31	110.52 ± 30.58	126.31 ± 18.85^‡^	95.80 ± 30.06	118.74 ± 30.49^‡^
Atherogenic Index^**^	2.24 ± 0.89	1.93 ± 0.78	2.35 ± 0.83	2.70 ± 0.73	2.06 ± 0.65	3.12 ± 1.05^‡‡^

Values are presented as mean ± SD

*SSG* single-session group, *MSG* multiple-session group, *CTG* control group, *BMI* body mass index, *TC* total cholesterol, *LDL* low-density lipoprotein cholesterol

^*, **^ Significant interaction effect between time and group (*p* < .05 and *p* < .01, respectively)

^†^ Significant main effect of time (*p* < .05)

^‡, ‡‡^ Significantly different from pre-intervention value (*p* < .05 and *p* < .01, respectively)

## Discussion

It has been reported that lack of physical activity can lead to metabolic disorders, which may lead in turn to hyperlipidaemia, hyperinsulinemia, and arteriosclerosis; thus, lack of exercise is a major factor in raising mortality risk [33]. Accordingly, the American College of Sports Medicine [34] recommends that adults participate in at least 150 min/wk. of moderate-intensity physical activity. However, many people, although they may recognise the positive effects of exercise, do not exercise regularly owing to lack of time. The concept of intermittent exercise over several short sessions was introduced to increase the exercise rate among such people.

With regard to body composition, there were significant decreases in weight, BMI, and fat mass in the SSG, which was in line with the findings of Skyes et al. [35], who reported a reduction in weight and fat mass in middle-aged women who engaged in prolonged aerobic exercise sessions intended to burn 400 kcal a day. This finding was also in line with the results of Osei-Tutu and Campagna [36], who found no positive changes in obesity-related factors in middle-aged obese women who engaged in multiple short sessions of exercise for 8 weeks. However, 8 weeks may be too short a time for multiple short sessions to have an effect on such factors. Single prolonged exercise sessions along with a calorie-restricted diet thus seem more effective than multiple short sessions with a calorie-restricted in reducing fat mass and improving body composition in obese individuals. However, Alizadeh et al. [37] recently reported that multiple short sessions of exercise for 150 min or more per week were more effective in reducing weight among obese and overweight individuals than single prolonged sessions. Further studies are needed that focus more closely on the long-term effects of multiple short sessions of exercise on body composition.

The American College of Sports Medicine also gives importance to the primary prevention of metabolic syndrome and management of risk factors. In this aspect, regular aerobic exercise can decrease blood pressure, improve hyperlipidaemia and blood glucose sensitivity, and decrease cardiovascular disease, mortality, and morbidity [38]. Our study found a decrease in SBP and an increase in HDL in the SSG, as well as decreases in both glucose and waist circumference in the SSG and MSG. These findings are similar to those of Murphy et al. [39], who found positive changes in blood lipid and glucose levels in middle-aged overweight women after 3 sessions of 10-min exercise or 1 session of 30-min exercise a day. In addition, SBP and weight showed simultaneous decreases only in the SSG. This result corroborates the suggestion of Mertens and Van Gaal [40] that losing weight alone can lower blood pressure. Given these results, it seems that a single prolonged exercise session with a calorie-restricted diet may reduce the risk of metabolic syndrome to a greater degree than multiple short sessions with a calorie-restricted diet; however, the most effective approach remains unclear.

One clear result of this study was that multiple short sessions of exercise with a calorie-restricted diet were as effective as a single prolonged session with a calorie-restricted diet in reducing blood glucose and waist circumference. This finding is similar to that of Tjønna et al. [41], who reported a reduction in the blood glucose and waist circumference of patients with metabolic syndrome after 16 weeks of either continuous exercise or intermittent exercise. It is also supported by Gayda et al. [22] who reported similar patterns of lipid oxidation with both continuous exercise and intermittent exercise. It is thus deemed that intermittent exercise is as effective as continuous exercise for reducing blood glucose and waist circumference.

This study found increases in LDL in the MSG and the CTG and an increase in AI in the CTG, suggesting that a single prolonged exercise session is better for LDL levels than multiple short sessions. Accumulation of LDL inside the blood vessels serves as a major cause of arteriosclerosis. Regular exercise enhances the heart and vessel functions and can thus prevent or delay the progress of cardiovascular diseases, including coronary artery disease, in part by improving cholesterol levels [17, 42, 43]. However, unlike this study, a previous study produced reported a reduction of LDL after 3 months of regular exercise [44]. In our study, although all participants were obese, their LDL levels were normal from the start, meaning there was little chance for the exercise intervention to lower levels further. Our results suggest that multiple short sessions of exercise with a calorie-restricted diet are not enough to facilitate fat oxidation sufficiently to prevent LDL accumulation.

Decreased serum HDL levels are associated with a high incidence of CVD and worse prognosis [45]. Pharmaceutical therapy based on lifestyle modification plays an important role in dyslipidaemia treatment, but it is accompanied by residual risks and potential adverse effects [46]. However, regular exercise increases serum HDL levels [47]. This study found that HDL increased in the SSG, suggesting that a single prolonged exercise session with a calorie-restricted diet is better for HDL levels than multiple short sessions with a calorie-restricted diet.

To the best of our knowledge, this is the first study to statistically evaluate short-term changes in risk for metabolic syndrome and AI in middle-aged obese women by comparing single prolonged exercise and multiple short exercise sessions. However, the present study has several limitations. First, the sample size was too small for the findings to be generalised. Future research should include larger samples. Second, since the exercise programme lasted only 12 weeks, a longer training duration and higher frequency may result in further improvements in metabolic syndrome risk and AI in middle-aged obese women. Third, as a primary outcome, factors of metabolic syndrome and atherogenic index or insulin resistance may be more appropriate than the percent body fat suggested in this study.

There are many different opinions about the best type of exercise for improving the metabolism. According to the American College of Sports Medicine, Centers for Disease Control and Prevention, and National Institutes of Health, moderate exercise is recommended [19]. In our study, the amount of exercise was small, but the intensity of exercise was higher, at 70–85% of VO_2max_ in both the SSG and MSG, compared with the popular recommendation. Therefore, future studies should examine the effects of multiple short exercise sessions of different lengths and intensities.

## Conclusions

This study shows that over 12 weeks, both single prolonged and multiple short exercise sessions with equal energy deficits for both modes had positive influences on the risk for metabolic syndrome and the AI in middle-aged obese women. Single prolonged sessions with a calorie-restricted diet appeared better for improving the risk of metabolic syndrome and atherosclerosis, but multiple short sessions with a calorie-restricted diet can effectively reduce blood glucose and waist circumference.

## Abbreviations

AI: Atherogenic index
BMI: Body mass index
BP: Blood pressure
CTG: Control group
DBP: Diastolic blood pressure
HDL: High-density lipoprotein
LDL: Low-density lipoprotein
MSG: Multiple-session group
SBP: Systolic blood pressure
SSG: Single-session group
TG: Triglyceride
